# Experts’ assessments of migration scenarios between the Middle East & North Africa and Europe

**DOI:** 10.1038/s41597-023-02532-1

**Published:** 2023-09-20

**Authors:** Michaël Boissonneault, Rafael Costa

**Affiliations:** 1 Netherlands Interdisciplinary Demographic Institute (NIDI-KNAW)/University of Groningen, The Hague, the Netherlands; 2 Department of Linguistics and Philology, Uppsala University, Uppsala, Sweden

**Keywords:** Society, Government, Law, Geography, Developing world

## Abstract

We describe data collected among 138 migration experts about the repercussions of scenarios of social change on migration between the Middle East & North Africa and Europe, during the period 2021–2030. Scenarios include changes in the cultural, demographic, economic, and political determinants of migration in sending and receiving countries. Assessments focus on the change in the number of family, work, and return migrants, the number of refugees, and the likelihood of achieving safe, orderly, and regular migration. Experts were at the moment of the survey active in European research centers or European (supra-) national governmental or civil society organizations. The survey features a factorial design, which allows for identifying a causal relationship between the experts’ assessments and the scenarios of social change. Our data may be used to estimate projection models of future migration flows, map out what experts consider as critical migration issues for the region, and identify areas of agreement or disagreement between them. As such, our dataset may illuminate decision-making regarding migration policies in Europe and beyond.

## Background & Summary

Migration to Europe from third-party countries is not only shaping more and more the continent’s demographic composition; it is also becoming a topic of increased political significance^1,2^. The aftermath of the 2015 refugee crisis has intensified political debates surrounding migration, with concerns arising over the European Union’s preparedness to manage large and sudden influxes of immigrants^3^. Recognizing the need to enhance Europe’s readiness in the face of potential migration waves, the European Commission initiated a research program in 2018 that aims to advance tools that enable the formulation of mid- and long-term migration scenarios specific to Europe^4,5^. This data descriptor contributes to this ongoing research effort by presenting data on the impact of scenarios of social change on migration from the Middle East and Northern Africa to Europe in the year 2030.

The methods used to generate the data presented here build on recent research that aimed at conciliating two approaches for exploring the future of migration^6,7^. In the first approach, quantitative models have been used that exploit past migration trends to make predictions about future levels between two regions. In this approach, scenarios are relied upon to assess the sensitivity of the models to different assumptions and convey the uncertainty of their outcomes^8^. In the second approach, the future of migration has been explored by means of qualitative storylines. Using this approach, scenarios were developed that correspond to different developments alongside preestablished dimensions. For example, storylines were developed which describe migration to Europe under different levels of economic development and different levels of cooperation between countries^9–12^.

Recent research aimed at conciliating the two approaches by offering quantified storylines of the future of migration between two regions^6,7^. Quantitative approaches are bound by what appears in the data record and do not allow to explore the impact of unprecedented events on migration. Yet, observers have argued in favor of considering such events as migration has proven amenable to relatively rare yet powerful events such as wars or economic downturns^12^. Quantitative approaches are furthermore less apt at incorporating the effect of complex mechanisms on migration such as tradeoffs and feedback loops, though they play a vital role in migration processes^13^. On the other hand, while qualitative storylines may consider the impact of unprecedented events and complex mechanisms in their scenarios, the difficulty remains of transforming narratives into quantified estimates of migration flows.

The problem of deriving quantities from qualitative scenarios has been so far solved by relying on expert surveys. In these, experts are presented with storylines that depict scenarios of social and environmental change in two regions and asked to quantify their impact on migration between them. One area that has received less attention, however, concerns how to collect assessments that quantify the effect of specific migration drivers while accounting for the effect of other drivers simultaneously, including tradeoffs and feedbacks. Indeed, studies have only been in state of considering either the effect of multiple drivers simultaneously (without quantifying the effect specific to each)^7^, or the one of single drivers in isolation from each other^6^.

It is with the goal of addressing this gap that the QuantMig survey was launched in the fall of 2021. The survey made use of a factorial survey methodology^14^ to derive from qualitative storylines quantities that can be traced back to specific drivers while simultaneously accounting for the effect of other drivers. Following this methodology, vignettes were presented to migration experts which depicted hypothetical changes in a series of social dimensions. Systematically changing the directions of change within each dimension and asking the experts to evaluate different vignettes made it possible to identify a causal impact between the change in the vignettes’ content and the variation in the experts’ assessments. This allowed us to obtain estimates of migration flows that are both linkable to specific changes in the underlying drivers while accounting for trade-offs and feedback mechanisms among them.

In this study, the vignettes’ content focused on the cultural, demographic, economic, and political determinants of migration. The choice of these four determinants was inspired by existing theories of social change^15^, and the nature of the changes within these determinants was based on previous reviews of migration drivers^16,17^. Throughout the survey, reference was made to Europe as receiving countries and to the countries of the Middle East and North Africa (MENA) as sending countries. Both the scenarios and their eventual impact on migration referred to the period included between 2021 and 2030, and assessments concerned changes in four types of migration flows (family, work, asylum, and return migration) as well as the likely repercussions on the likelihood of achieving safe, orderly, and regular migration as stipulated in the Global Compact for Migration^18^.

Opinions were sought among experts active in European research centers and universities, as well as in European (supra) governmental organizations and organizations of the civil society. As such, the QuantMig survey stands out not only for the large number of experts from which it solicited input (138 of them), but also for its striving toward including the opinion of experts active in civil society organizations, whereas previous expert surveys mainly considered experts active in academia and working for governmental agencies.

The survey data may be used in combination with data on past migration flows to estimate projection models of future migration flows between MENA countries and Europe, but also to map out what experts consider as critical migration issues for the region and to identify areas of agreement or disagreement between them. As such, we believe that the dataset we present here has the potential to illuminate decision-making regarding migration policies in Europe and beyond.

## Methods

This section describes the methods employed to collect data among migration experts. A more elaborate discussion of the motivations behind these methods is provided in reference^19^.

### Scope

We referred in our survey to Europe as the 27 member states of the European Union (as of 2021), the four member states of the European Free Trade Association (Norway, Iceland, Lichtenstein, Switzerland), and the United Kingdom. We referred to MENA countries as the countries of northern Africa (Algeria, Morocco, Libya, Egypt, Tunisia), the Arabian Peninsula (Kuwait, Saudi Arabia, Oman, Yemen, Bahrein, United Arab Emirates, Qatar), the Levant (Israel, the State of Palestine, Jordan, Lebanon, Syria), the Caucasus (Armenia, Azerbaijan, Georgia), Central Asia (Kazakhstan, Uzbekistan, Turkmenistan, Tajikistan), as well as to Iran and Iraq.

We asked respondents to evaluate the consequences of social change for migration in the period 2021–2030, using the level of migration in the year 2019 as a reference. We instructed respondents to disregard the impact that the COVID-19 pandemic was having on migration at the moment of the survey as we hypothesized that this impact would have resorbed by 2030.

### Vignettes

**Fig. 1:**
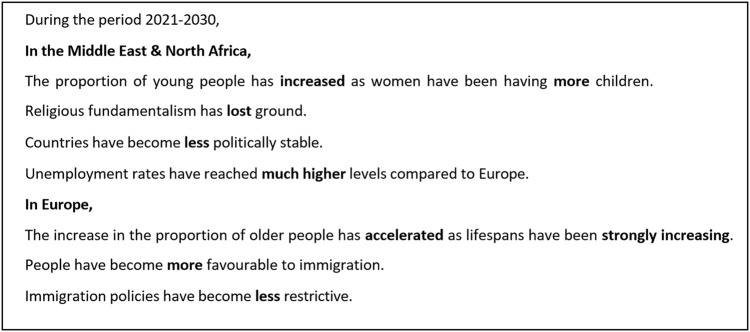
Vignette example. Each line contains a statement about possible social changes within the demographic, cultural, political, and economic domains, in Europe and MENA countries. Text in bold was systematically changed between vignettes. In half of the vignette versions, the order of presentation of the statements was inverted (lines 2 to 6 were inverted with lines 7 to 10).

The vignettes covered four of the five dimensions of social change considered in de Haas *et al*.’s theory of social transformation (the cultural, demographic, economic, and the political, dimensions), excluding the technological dimension as we considered that there was no specific changes in this dimension that would have a straightforward impact on migration in the region^15^. Based on reviews of the determinants of migration, we elaborated for the cultural, demographic, and political dimensions two diametrically opposite scenarios of change separately for Europe and the MENA countries. As for the economic dimension, we developed a scenario of change common to the two groups. The vignette universe thus included 128 distinct vignettes resulting from the combination of seven factors with two levels each (2^7^ = 128). The seven factors and their levels are specified in Table 1 while Fig. 1 shows an example of a vignette.

**Table 1:** Vignette factors and their corresponding levels, for each of the four selected domains, for MENA countries and Europe.

Domains	Levels	Factors	
		MENA countries	Europe
Demographic	Increase/decrease in…	…the proportion of young people.	…the pace of population aging.
Cultural		…the level of fundamentalism.	…people’s favorability to immigration.
Political		…the level of political stability.	…the level of immigration policy restrictiveness.
Economic		…the level of convergence in unemployment

### The experimental design

Respondents were asked to evaluate four vignettes. This number appeared as a good compromise between maximizing the total number of vignette evaluations and maintaining a reasonable response burden. The four vignettes per questionnaire meant that we needed 32 distinct questionnaire versions to cover our entire vignette universe. For each questionnaire version, vignettes were presented to participants following one of two orders: the “MENA first order”, with the demographic, cultural, and political factors in MENA countries coming first, followed by the economic factor, followed by the demographic, cultural, and political factors in European countries; and the “Europe first order”, with the demographic, cultural, and political factors in European countries coming first, followed by the cultural and political factors in MENA countries, followed by the economic factor. We used the command *optBlock* of the R package AlgDesign to generate the vignettes and assign them to the different questionnaire versions^20^.

### The measures

Respondents were asked to evaluate the changes implied by the content of the vignettes for four migration flows: family, work, refugee, and return migration. Respondents were provided with the following explanations about each type of migration. Family and work migrants referred to migrants who are granted a residence permit by their host country for family or professional reasons, respectively. Refugees are those who are granted the status of refugees or temporary protection, while return migrants are Middle Eastern and North African nationals who return to their country of origin after having been granted a residence permit (of any kind) in a European country. According to these instructions, respondents were not to consider irregular migration in their answers.

Respondents were asked to express the change in each migration flow using a scale that included the following values: multiplication by a factor of 1.25, 1.5, 2, 3, or 5; a division by the same factors; or no change. Each assessment was made using a slider that participants could move along the response scale. As respondents moved the slider, a graph showed the implied change in the number of migrants in the year 2030 and compared it with past migration levels for the period 2010–2019 as provided in the Eurostat database on residence permits (concerning family migrants, work migrants, and refugees^21^) and previously released QuantMig data (concerning return migrants)^22^.

Respondents were further asked to evaluate the change implied by the content of the vignettes for the likelihood of achieving safe, orderly, and regular migration. “Safe, orderly, and regular migration” referred to the set of 23 objectives listed in the Global Compact for Migration (United Nations, 2017)^18^. Respondents were allowed to express the change in this likelihood by selecting one of the following answers: “Much more difficult”, “Moderately more difficult”, “Somewhat more difficult”, “Neither more nor less difficult”, “Somewhat less difficult”, “Moderately less difficult”, “Much less difficult”.

### The survey

#### Implementation

The survey was implemented online. Participants could connect with the webpage hosting the questionnaire by clicking on the URL included in the invitations we sent. Responses from fully completed questionnaires were saved anonymously to a database that we (the authors) could only access using a secret password. Any attempt at terminating the connection with the webpage without having filled out the whole questionnaire would result in an error message. Responses from respondents who would nevertheless terminate the connection with the site were automatically discarded.

Upon each connection with the survey’s webpage, a new questionnaire was selected from our set of 64 predefined questionnaires. The selection of a questionnaire was made based on an algorithm that identified in our database the questionnaire version that had been completed the least often at the time the connection was established. If an additional connection occurred at the same time, the algorithm would identify the next questionnaire with the fewest number of answers, and so on as far as multiple connections would happen simultaneously.

#### Structure

Respondents were asked to answer a total of fourteen questions. In questions 1 to 5, respondents were asked about their professional experience with migration and background. In questions 6 to 8, they were asked to provide an evaluation of the number of migrants in the year 2030 as well as of the likelihood of achieving safe, orderly, and regular migration supposing a continuation of the underlying trends in Europe and MENA countries. Respondents were furthermore asked how confident they felt that their assessment of the number of migrants in the year 2030 would lie closer to the actual value compared to any other value on the scale. This part of the survey aimed at evaluating the heterogeneity in response behavior among participants, without the influence of the vignettes.

With questions 9 to 13, we asked respondents to provide estimates of the change in the number of family migrants, work migrants, refugees, and return migrants, and an estimate of the change in the likelihood to achieve safe, orderly, and regular migration resulting from the vignettes’ content. This set of questions was repeated four times (once after each vignette).

The last question was open-ended and enjoined participants to indicate whether they believed that factors other than those included in the vignettes could play an equally important role in driving future migration flows between MENA countries and Europe. Table 2 lists all the questions contained in the questionnaire and the corresponding response scales. A version of the full questionnaire is supplied in reference^19^.

**Table 2:** Survey questions and corresponding response scales.

#	Text	Values
1	In your profession, do you often think about the future of migration?	No, Never; Yes, Sometimes; Yes, Most of the time
2	How familiar are you with the way that structural factors influence people in their decision to migrate?	Not familiar at all; Somewhat familiar; Moderately familiar; Considerably familiar
3	What is the highest level of education you have completed?	Secondary education; Post-secondary education; Bachelor’s; Master’s; Doctorate
4	To which sector does your employer belong?	Academia; Civil society; Government; Other
5	For how many years have you been working on issues relating to migration?	Any natural number.
6	Supposing a continuation of the demographic, economic, cultural and political trends in Europe and the Middle East & North Africa. Compared to 2019, the total number of migrants from the Middle East & North Africa to Europe will be in 2030…	Divided by 5, 3, 2, 1 1/2, 1 1/4, No change, Multiplied by 1¼, 1 ½, 2, 3, 5
7	How confident are you that the actual number of migrants in 2030 will lie closer to the value you chose (compared to any of the other proposed values)?	50% confident; 75% confident; 90% confident; 95% confident
8	Supposing a continuation of the demographic, economic, cultural and political trends in Europe and the Middle East & North Africa. How difficult will it be to achieve safe, orderly and regular migration between Europe and the Middle East & North Africa by the year 2030, as stipulated in the Global Compact for Migration?	Very difficult; Moderately dif.; Somewhat dif.; Not particularly easy or dif.; Somewhat easy; Moderatly easy; Very easy
9	Based on [the situation described by the vignette], compared to 2019, the number of family migrants from the Middle East & North Africa to Europe will be in 2030…	Same as in Question 6
10	Based on [the situation described by the vignette], compared to 2019, the number of work migrants from the Middle East & North Africa to Europe will be in 2030…	Same as in Question 6
11	Based on [the situation described by the vignette], compared to 2019, the number of refugees from the Middle East & North Africa to Europe will be in 2030…	Same as in Question 6
12	Based on [the situation described by the vignette], compared to 2019, the number of return migrants from the Middle East & North Africa to Europe will be in 2030…	Same as in Question 6
13	Based on [the situation described by the vignette], compared to 2019, do you believe that it will be more or less difficult to achieve by the year 2030 safe, orderly and regular migration?	Much more difficult; Moderately m. dif.; Somewhat m. dif.; Neither m. or less dif.; Somewhat l. dif.; Moderately l. dif.; Much l. dif.
14	Indicate whether you believe that there are factors that will have an impact on migration between Europe and the Middle East & North Africa that is equally large or larger than those referred to in this study. If so, indicate which ones. You may further provide any comment that you may have on any other aspect of the survey.	Open-ended

#### Pilots

We ran two pilots before launching the survey. The first one took place on 30 June 2021 among three migration scholars based at the Netherlands Interdisciplinary Demographic Institute in The Hague (NIDI-KNAW), the Netherlands. Participants received the PDF version of the questionnaire and were asked to answer questions with pen and paper. The second pilot took place between 25 October and 4 November 2021 using the web version of the survey. Four migration scholars involved in the QuantMig project and based in different research institutes across Europe participated and provided feedback about the vignette text and the graphs. Finally, one researcher in psychology with experience with factorial survey experiments provided feedback about the methodology and the survey content.

#### Recruitment

The population of interest consisted of migration professionals working for European academic, governmental, and civil society organizations. Participants were selected as candidate respondents via a list of European organizations that we established prior to launching the survey. This list included the organizations listed by the national contact points of the European Migration Network (EMN), the organizations part of the International Migration Research Network (IMISCOE), the European national offices of the International Organization for Migration (IOM), and the national statistical institute of each European country. Organizations listed by the national contact points of the EMN were found on the website of each of these contact points or, if unavailable, were provided to us after we had requested them via email or an online form. The organizations part of IMISCOE and the IOM were found on these networks’ respective websites, while the statistical institutes were identified using the Google search engine. This process allowed us to make an initial list of 233 organizations.

For each of the organizations in our list, we retrieved the email addresses of at least one person, usually the director or the head of the migration lab, and sent them an email in which we enjoined them to participate in our survey. If for a given organization we could not find the email address of any candidate participants, we sent a general query to ask about the names and email addresses of candidate participants whom we then contacted directly. In each email, we also enjoined candidate participant to share the survey’s URL with any of their colleagues who might be interested in participating. As part of this step, we contacted a total of 258 potential participants.

In the second step, we contacted scholars listed on the website of the “Council for migration” (*Rat für Migration*), an association of approximately 150 Germany-based researchers working on migration from different perspectives, as well as a number of the authors’ contacts who work on migration. This last step allowed us to contact an additional 161 potential participants.

The first invitations were sent on 24 November 2021 (the first day that the questionnaire was available online). Potential participants who did not reply to our first email were contacted again one week following the initial contact, and those who did not reply to any of our first two emails were contacted a third time two weeks following the initial contact. The last invitations were sent on 18 January 2022 and the website closed on 4 February 2022. At this point, there had been 841 connections established with the website and 138 questionnaires had been fully completed.

### Ethical approval, respondents’ privacy, and protocol registration

We consulted the ethics committee of the Faculty of Behavioural and Social Sciences at the University of Groningen to determine whether ethical approval was needed for our survey. The committee provided ethical clearance without further evaluation as we were interested in the professional judgment of the participants rather than their personal one. To preserve the respondents’ anonymity, we refrained from asking them for any personal information. We informed each respondent that their responses would be used for the study only and that all information would remain anonymous given it would be transferred to a database with random identifying numbers. They were finally informed that they could quit at any time while answering the survey and that their responses would only be saved upon completing the whole survey. The survey protocol was registered on 23 November 2021 in the Open Science Framework^23^.

## Data Records

We produced two databases, both of which are deposited on Zenodo^24^. The first one (‘QMsurvey_respondent.xlsx’) contains responses to questions 1–8 and 14. In this database, each row corresponds to one respondent. The second database (‘QMsurvey_respondentvignette.xlsx’) contains responses to questions 9–13. Here, each row corresponds to one vignette (i.e. four lines for each respondent). Both databases contain generated variables that we created to help navigate the data. Finally, the database *QMsurvey_respondentvignette.xlsx* contains variables that summarize the content of the vignettes used to elicit responses from the respondents; these can for example be used as predictors in analyses. The two databases may be used in combination or on their own. For example, analysts interested only in the effect of the vignettes’ content on the respondents’ assessments may use the *QMsurvey_responentvignette.xlsx* database. In contrast, analysts interested in the effect of the respondents’ characteristics on the assessments need to merge the two databases based on the ID variable included in both. Codebooks are available online for both databases^25^.

## Technical Validation

This section first discusses the expert sample and then concentrates on the vignette assessments.

### The sample

Although the recruitment process specifically targeted migration experts, in practice, anybody could access the survey and fill in the questionnaire. Considering this, questions 1 and 2 aimed at measuring the degree of familiarity of the respondents with the topic of the survey. Responses indicate that all respondents thought at least sometimes about the future of migration within their professional activities and that all were at least somewhat familiar with the structural factors that compel people to migrate (first two columns, Supplementary Information).

One of the goals of the QuantMig survey was to collect opinions about the future of European migration not only from academics or analysts employed by statistical agencies (as this has so far been the case in previous expert surveys on migration^6,7^) but also from people working for organizations part of civil society or other non-governmental agencies. This goal was partly met. While we did obtain participation from professionals working in civil society organizations or other (primarily private) organizations, these represent only a small fraction of the total sample (11%). Meanwhile, academics account for more than 50% of the sample.

The sample shows a fairly balanced profile between respondents with or without a doctorate degree as well as with respect to their number of years working on migration. Unfortunately, we could not include a breakdown of the respondents according to the country in which they were active at the moment of taking the survey as we did not include a question to this effect in the survey. However, our target population included only experts active in European research centers, universities, government agencies, and civil society organizations. As such, invitations were only sent to experts who were at the moment of the survey active in such organizations. Furthermore, although we made important efforts toward including all organizations within Europe that deal with migration, it is impossible for us to determine the range of possible views on migration that our survey effectively allowed to cover. For example, it cannot be excluded that experts adhering to specific views were more likely to participate than experts who share other views. Despite this, as we shall see in the next subsection, our survey did allow to generate a fair amount of variation in the assessments of future migration flows.

### The vignette assessments

Relying on the judgment of a large pool of experts allows for the measurement of the extent to which views on migration may differ. As shown in Supplementary Information (columns 3 to 10), assessments tend to vary not only between individuals in different groups, but also between individuals within the same group (as indicated by the standard deviation).

Data needed to be collected among a sufficiently large number of respondents to ensure that responses covered the whole vignette universe. Furthermore, multiple responses were needed for each questionnaire version to ensure that the effect of the variation in the vignettes’ content was not confounded with the one of the variation in response behavior between respondents. Prior to launching the survey, we aimed at achieving five full participations per questionnaire version, which would have required a total of at least 170 participations. Upon reaching the end of the period we had planned for our data collection, we had obtained five full participations for eleven questionnaire versions and four for twenty of them. We nonetheless deemed this outcome satisfying as previous authors pointed out that three participations by questionnaire version is usually sufficient for avoiding confoundedness between the effect of vignettes and the one of individual response behavior^14^.

To assess the suitability of our data for making predictions about the future of migration under scenarios of social changes, we ran random-intercept models with the expert assessments on each of the five migration outcomes as outcome variable and the seven vignette dimensions (reconverted into binary variables) as predictors. In each model, the participant identifying number was included as a grouping variable. Also, values for the number of family, work, refugee, and return migrants were converted into their logarithmic values since the response scales we used implied larger increases (or decreases) in the number of migrants as answers lied further away from the “no change” value.

Estimates were fairly precise as the standard errors associated with each predictor variable were considerably low for most outcomes, reaching values generally as low as 0.03 (Table 3). This was somewhat less true in the case of the assessments on the likelihood to achieve safe, orderly, and regular migration, where predictors had standard errors of about 0.1.

**Table 3:** Coefficient estimates and standard errors (between parentheses) for the five migration outcomes and the seven predictor variables included in the linear random-intercept models.

	Family migrants	Work migrants	Refugees	Return Migrants	Safe, orderly, regular migration
Intercept	−0.133 (0.04)	−0.064 (0.045)	−0.164 (0.054)	0.188 (0.043)	−0.506 (0.155)
**MENA countries**					
Increasing prop. young people	0.123 (0.025)	0.103 (0.03)	0.059 (0.035)	−0.034 (0.028)	−0.182 (0.103)
Increase in fundamentalism	0.054 (0.025)	0.044 (0.029)	0.179 (0.034)	−0.083 (0.028)	−0.302 (0.102)
Decrease in political stability	0.137 (0.025)	0.113 (0.03)	0.29 (0.034)	−0.126 (0.028)	−0.575 (0.103)
**MENA vs. EU**					
Increase in unemployment levels	0.12 (0.024)	0.176 (0.029)	0.162 (0.033)	−0.174 (0.027)	−0.422 (0.100)
**EU**					
Increase in pace of pop. aging	0.006 (0.025)	0.084 (0.029)	0.000 (0.034)	0.004 (0.028)	−0.038 (0.102)
Increase in favorability to immig.	0.114 (0.025)	0.115 (0.029)	0.009 (0.034)	−0.005 (0.028)	0.423 (0.102)
Decrease in policy restrictiveness	0.146 (0.025)	0.15 (0.03)	0.087 (0.034)	0.013 (0.028)	0.557 (0.103)

The dependent variable in the Family migrants, Work migrants, Refugees, and Return migrants models is the logarithm of the relative change in the number of migrants.

An important validity criterion of our survey was whether the variation in the assessments was indeed due to variation in the vignettes’ contents, rather than to variation between the participants’ response behavior. To answer this question, we estimated for each outcome an empty random intercept model including the id variable as a grouping variable. The resulting estimates, shown in Table 4, suggest that between two-thirds and three-quarters of the assessments’ variance can be explained by the vignettes’ contents, rather than by the variation in the respondents’ behavior.

**Table 4:** Amount of variance attributable to the variable ID and residual variance, random-intercept models, for each assessment type.

Type	Variance due to ID	Residual variance
Family	0.86	1.73
Work	0.76	2.20
Refugee	1.27	3.04
Return	0.62	2.01
Compact	0.50	1.61

**Fig. 2:**
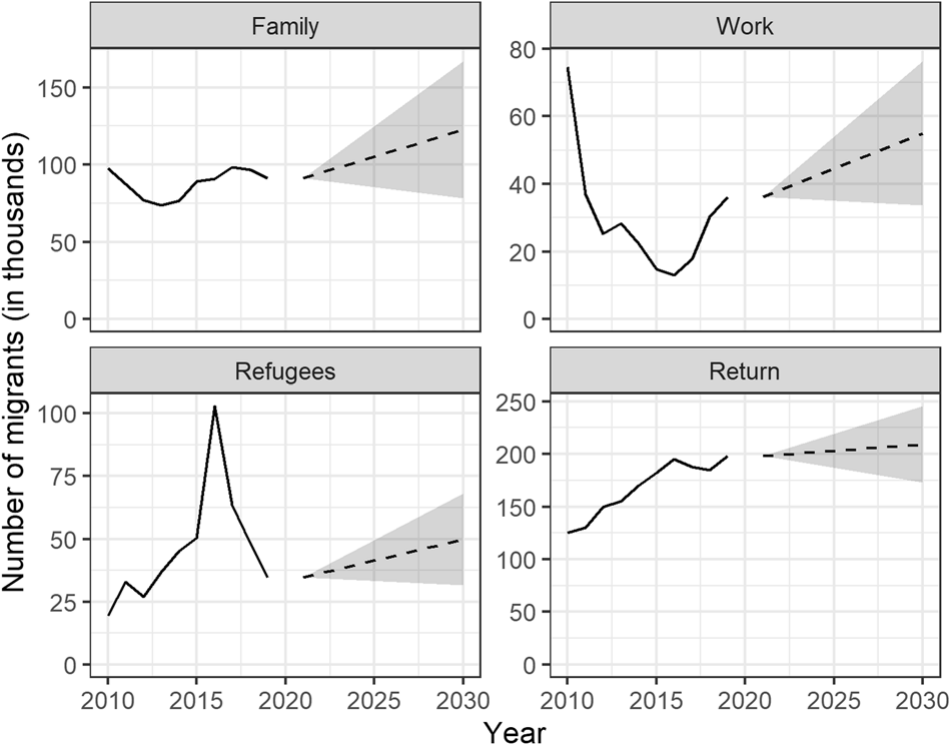
Past trends in the number of family, work, refugee, and return migrants to Europe from MENA countries (solid lines), mean assessments of future trends (dashed lines), and values for opposite scenarios of change in the cultural, demographic, economic & political determinants of migration (upper and lower limits, shaded area).

One possible use of the data is in projection models of migration. Though we obviously cannot determine whether the experts’ predictions will turn out to be accurate or not, we can gauge the plausibility of the experts’ assessments by aggregating their answers and comparing them with past trends. Fig. 2 gives an overview of these past trends and put them in relation with the mean values (see the dashed lines in Fig. 2) obtained across all vignette assessments, for each migration flow. These mean values can be interpreted as the respondents’ beliefs supposing a continuation of the current trends in the cultural, demographic, economic, and political dimensions affecting migration between MENA countries and Europe over the period 2021–2030.

By design, the QuantMig survey allows for an assessment of the factors that may influence an expert when evaluating future migration trends. To explore by how much assessments could vary depending on changes in the content of the vignettes, we designed here two scenarios of social change in MENA countries and Europe that took opposite values in each of the seven dimensions included in the survey. More specifically, in one scenario, we considered a situation where in MENA countries (1) The proportion of young people has increased; (2) Religious fundamentalism has gained ground; (3) Countries have become less politically stable; and (4) Unemployment rates have reached much higher levels compared to Europe. Meanwhile, in the same scenario, we considered in Europe an (5) Acceleration in the increase in the proportion of older people; (6) A greater favorability of public opinion toward immigration; and (7) Less restrictive immigration policies. By summing the regression estimates specific to each outcome, we obtained what can be interpreted as an upper value for the number of people migrating from MENA countries to Europe, i.e. one that would be reached if all the conditions were in place to stimulate migration between the two regions. This value corresponds to the upper limit of the shaded areas in Fig. 2.

We repeated the same procedure this time taking the regression estimates corresponding to the alternate outcomes in each of the seven predictors (which turned out to correspond to the intercepts’ values). The resulting value can be interpreted as the number of people who, according to our sample of experts, would migrate if all the conditions were in place to deter migration between the two regions and corresponds to the lower limit of the shaded areas in Fig. 2. Clearly, the vignettes’ contents successfully induced variation in the respondents’ assessments; however, whether these will prove to be accurate estimates of future migration flows will become clear in only a number of years.

## Usage Notes

As a usage note we recommend the use of linear regression models with random intercepts and fixed means for the analysis of the effect of the vignette contents on the respondents’ assessments. Doing so, analysts should include the id variable contained in the databases as a grouping factor in the regression formula. It is also recommended to transform the values for the number of family, work, refugee, and return migrants into their logarithmic values prior to estimating these models.

### Supplementary information

Supplementary Information

## Data Availability

The R code used to prepare the survey and produce the tables and figures presented here is available at https://github.com/michaelboissonneault/quantmigsurveycode-eurostatdata.
